# Loss of hypothalamic MCH decreases food intake in amyotrophic lateral sclerosis

**DOI:** 10.1007/s00401-023-02569-x

**Published:** 2023-04-14

**Authors:** Matei Bolborea, Pauline Vercruysse, Tselmen Daria, Johanna C. Reiners, Najwa Ouali Alami, Simon J. Guillot, Stéphane Dieterlé, Jérôme Sinniger, Jelena Scekic-Zahirovic, Amela Londo, Hippolyte Arcay, Marc-Antoine Goy, Claudia Nelson de Tapia, Dietmar R. Thal, Kazumoto Shibuya, Ryo Otani, Kimihito Arai, Satoshi Kuwabara, Albert C. Ludolph, Francesco Roselli, Deniz Yilmazer-Hanke, Luc Dupuis

**Affiliations:** 1 Université de Strasbourg, INSERM, Mécanismes centraux et périphériques de la neurodégénérescence, UMR-S1118, Strasbourg, France; 2 School of Life Sciences, University of Warwick, Gibbet Hill Road, Coventry, CV4 7AL UK; 3 Clinical Neuroanatomy Section, Department of Neurology, Ulm University, Ulm, Germany; 4 Institute for Neurobiochemistry, Ulm University, Ulm, Germany; 5 Department of Neurology, Neurology Clinic, Ulm University, Ulm, Germany; 6 Laboratory for Neuropathology, Institute for Pathology, Ulm University, Ulm, Germany; 7 Laboratory for Neuropathology, Department of Imaging and Pathology, and Leuven Brain Institute, KU louvain, Belgium; 8 Department of Pathology, UZ Leuven, Japan; 9 Department of Neurology, Chiba University School of Medicine, Chiba, Japan; 10 Deutsches Zentrum für Neurodegenerative Erkrankungen (DZNE), Ulm, Germany

## Abstract

Amyotrophic lateral sclerosis (ALS) is associated with impaired energy metabolism, including weight loss and decreased appetite which are negatively correlated with survival. Neural mechanisms underlying metabolic impairment in ALS remain unknown. ALS patients and presymptomatic gene carriers have early hypothalamic atrophy. The lateral hypothalamic area (LHA) controls metabolic homeostasis through the secretion of neuropeptides such as orexin/hypocretin and melanin-concentrating hormone (MCH). Here, we show loss of MCH-positive neurons in three mouse models of ALS based on *SOD1* or *FUS* mutations. Supplementation with MCH (1.2 µg/d) through continuous intracerebroventricular delivery led to weight gain in male mutant *Sod1*^*G86R*^ mice. MCH supplementation increased food intake, rescued expression of the key appetite-related neuropeptide AgRP (agouti-related protein) and modified respiratory exchange ratio, suggesting increased carbohydrate usage during the inactive phase. Importantly, we document pTDP-43 pathology and neurodegeneration in the LHA of sporadic ALS patients. Neuronal cell loss was associated with pTDP-43-positive inclusions and signs of neurodegeneration in MCH-positive neurons. These results suggest that hypothalamic MCH is lost in ALS and contributes to the metabolic changes, including weight loss and decreased appetite.

**Supplementary Information:**

The online version contains supplementary material available at 10.1007/s00401-023-02569-x.

## Introduction

Amyotrophic lateral sclerosis (ALS), also called Lou Gehrig’s disease or Charcot’s disease, is a rapidly progressive and invariably fatal neurological disease affecting motor neurons. The average life expectancy after the diagnosis is approximately 2 years, making it one of the most fatal neurological diseases associated with the highest unmet medical needs for prevention and treatment.

Motor symptoms of ALS are frequently accompanied by weight loss, which often appears up to 15 years before motor symptom onset as indicated by epidemiological register studies [55, 76] as well as population-wide prospective cohort studies [32, 52, 55]. Weight loss is a negative prognostic marker, and correlates with a faster progression of motor impairment [18, 44, 53], whereas higher levels of blood lipids have been associated with prolonged survival [21, 23]. Counteracting weight loss by increasing the energy content of diet raised survival rates in ALS mouse models [24], and recent clinical trials supported the potential translational relevance of these studies. Indeed, hypercaloric nutrition increased survival in gastrostomized ALS patients [20, 77]. Also, ALS patients with normal to fast disease progression showed benefits from high-caloric dietary supplementation in our recent LIPCAL-ALS trial [41]. Serum neurofilaments, a marker of axonal injury, also decreased after dietary intervention in the same trial [22]. Thus, the identification of the biological basis of weight loss could provide possible therapeutic targets in ALS.

Early weight loss in ALS patients appears mechanistically complex, and could be caused by both changes in energy expenditure [16–19, 30] and loss of appetite [51]. These mechanisms are both centrally integrated into the hypothalamus [48, 78] through a complex network of neuropeptidergic neurons and glial cells [48, 78], and increased fiber density positive for Agouti-related protein (AgRP), a powerful orexigenic peptide [66, 73]. Recent work highlighted that the lateral hypothalamic area (LHA), a region critical in feeding, drinking, stress, motivational behavior, locomotion, and arousal/sleep, could be affected in ALS [14, 31]. Consistently, the LHA connectome is modified in ALS mouse models [3]. While the area contains about 30 subtypes of neurons involved in various LHA functions [6, 46], melanin-concentrating hormone (MCH) neurons in this region that are crucial for the regulation of metabolism remain unexplored in the context of the disease.

Here, we investigated whether MCH neurons in the LHA undergo neurodegeneration in both ALS patients and mouse models of ALS. At a functional level, we further examined whether this loss contributes to metabolic alterations observed in ALS mouse models and whether intracerebroventricular (i.c.v.) MCH delivery can rescue such alterations.

## Materials and methods

### Human subjects and neuropathological evaluation of the brain

Autopsy tissue from ALS cases (*n* = 17, 52.9% female, age: 62.6 ± 2.5 years [mean ± S.E.M.]) was obtained from a study cohort previously described in detail and used upon approval by the University of Chiba Ethical Committee [12]. Thirteen randomly selected controls without a history of neurodegenerative disease (*n* = 13, 30.8% female, age: 65.9 ± 3.5 years [mean ± S.E.M.]) collected at the Clinical Neuroanatomy Section of the Department of Neurology (University of Ulm, Germany) were included for comparison with ALS cases. All autopsied subjects underwent routine neuropathological examination and were screened for Alzheimer’s disease (AD)-related neurofibrillary tau pathology [8], other tauopathies, the Parkinson’s disease (PD) stage based on alpha-synuclein pathology [11], other alpha-synucleinopathies, and the beta-amyloid (Aβ) phase [70]. The ALS stage was determined based on the extent of phosphorylated-TAR DNA-binding protein 43 kDA (pTDP-43) pathology in the brain and spinal cord [9, 13]. Demographics, clinical data, and neuropathological assessment of neurodegenerative stages for the patient cohort used for the analysis of hypothalamic pathology are summarized in Table 1**.** This retrospective study was conducted in compliance with the University ethics committee guidelines (#19/12 and # 135/20) as well as the German federal and state law governing human tissue usage and in accordance with the Declaration of Helsinki. Informed written permission was obtained from all patients and/or their next of kin for autopsy.

**Table 1:** Demographics, clinical data, and neuropathological diagnosis of patients with ALS and controls included to the study

Case No	Group	Sex	Age	Disease duration	NFT stage (AT8)	Amyloid phase	PD Stage	ALS stage	Clinical diagnosis
1	ALS*^,#^	f	63	n.a	3	0	1	4	ALS
2	ALS*^,#^	f	56	60	2	0	0	4	ALS
3	ALS*	f	70	36	3	2	0	3	ALS
4	ALS*	f	64	36	3	0	0	2	ALS
5	ALS*	f	59	36	1	2	0	2	ALS
6	ALS*	f	40	48	2	0	0	3	ALS
7	ALS*	f	42	n.a	1b	0	0	2	ALS
8	ALS*	f	79	n.a	2	1	0	3	ALS
9	ALS*^,#^	f	67	12	3	0		4	ALS
10	ALS*^,#^	m	60	72	1	0	0	4	ALS
11	ALS*	m	67	144	3	2	0	4	ALS
12	ALS*^,#^	m	74	12	3	0	0	4	ALS
13	ALS*	m	66	48	1	2	0	3	ALS
14	ALS*^,#^	m	73	36	2	2	0	3	ALS
15	ALS*	m	62	24	3	0	0	2	ALS
16	ALS*	m	53	36	1b	0	0	2	ALS
17	ALS*	m	69	n.a	1	0	0	1	ALS
18	Control*^,#^	f	79	–	2	0	0	0	Ruptured aotic aneurysm
19	Control*^,#^	f	62	–	3	0	0	0	Stomach cancer, peritoneal carcinomatosis
20	Control^#^	f	63	–	3	0	0	0	Rectal adenocarcinoma
21	Control^#^	f	78	–	2	0	0	0	Cardiac arrhythmia with ventricular fibrillation
22	Control^#^	m	57	–	1	0	0	0	Renal atrophy, intestinal necrosis
23	Control^#^	m	46	–	1	0	0	0	Drug overdose
24	Control*^,#^	m	56	–	1	0	0	0	Metastatic lung adenocarcinoma, recurrent pulmonary embolism
25	Control*	m	54	–	1	0	0	0	Perforated stomach ulcer with bleeding
26	Control*^,#^	m	73	–	2	0	0	0	Myocardial infarction, chronic obstructive pulmonary disease (COPD)
27	Control *	m	71	–	2	0	0	0	Metastatic renal cancer (hypernephroma)
28	Control*	m	78	–	3	2	0	0	Myocardial infarction, coronary artery disease (CAD)
29	Control*	m	73	–	3	1	0	0	Bronchial asthma, aortic aneurysm, prostate hyperplasia, alcohol abuse, history of two alcohol withdrawal seizures
30	Control *	m	67	–	3	0	0	0	Metastatic urinary bladder carcinoma, chronic interstitial nephritis, pulmonary emphysema

Mean age ± SD for ALS 62.6 ± 10.4 and Controls 65.9 ± 10.5; 52.9% females in ALS and 30.8% females in Controls. f: female; m: male; n.a.: not available; SD: standard deviation

^*^Quantitative analyses in the PN (pigment Nissl) stain

^#^Quantitative analyses of PMCH-positive neurons with and w/o pTDP43 aggregates

### Immunohistochemistry (IHC) and immunofluorescence (IF) in human LHA

Thick brain sections were cut at 70 µm from paraffin-embedded autopsy tissue using a sliding microtome (Jung, Heidelberg, Germany) as described previously [27]. For topographical orientation, free-floating sections were pre-treated with performic acid and processed with aldehyde fuchsin for selective staining of lipofuscin pigment combined with a basophilic Nissl stain (Darrow red) [7]. Antigen retrieval was performed by pre-treating the section with performic acid or in a steamer (95 °C) with 10 mM citrate buffer at pH 6.0 for 10–15 min. Endogenous peroxidase activity was inhibited with a mixture of 10% methanol and 3% concentrated H_2_O_2_ in Tris-buffered saline (TBS) for 30 min. Non-specific binding sites were blocked using 5% bovine serum albumin (BSA) for 30 min at room temperature. For the visualization of MCH neurons, sections were incubated with a primary antibody against pro-melanin-concentrating-hormone (PMCH) overnight at 4 °C (1:1000 polyclonal rabbit, HPA046055, Atlas Antibodies AB, Bromma, Sweden). The levels of PMCH in the LHA are expected to correspond to the expression of MCH, as PMCH prohormone is cleaved into MCH and neuropeptide glutamic acid-isoleucine (NEI) peptides, both expressed within the same neurons in human hypothalamus [28]. The expression of neuropeptide-glycine-glutamic acid (NGE), which is a third mature peptide derived from the prohormone PMCH, has not been observed in the brain [74]. ALS-related pathological protein aggregates were detected by incubating the sections with the primary anti-pTDP-43 antibody, which detects TDP-43 abnormally phosphorylated at S409/410 (1:4000 for IF, 1:10000 for IHC, polyclonal rabbit, TIP-PTD-P02, Cosmo Bio Co., Ltd, Tokyo, Japan). For single- and double-label IHC, sections were incubated with a secondary biotinylated antibody (1:200; 2 h, room temperature, Vector Laboratories, Burlingame, CA, USA) followed by incubation with the avidin–biotin-peroxidase complex (1½ hours, room temperature, ABC Vectastain kit, Vector Laboratories, Burlingame, CA, USA). For the visualization of the first primary antibody, 3,3´-diaminobenzidine tetrahydrochloride (DAB; Sigma Taufkirchen, Germany) was used as the chromogen. For double-label IHC, sections were further treated with TBS at 95 °C for 5 min, and the whole immunohistochemical procedure was repeated using the next primary and secondary antibody. Subsequently, a blue chromogen (Vector SK-4700 peroxidase substrate kit, Linaris, Doffenheim; Germany) was used to visualize the second reaction product. Finally, tissue sections were cleared, mounted, and cover-slipped in a medium with a refraction index of 1.58 (Histomount, Thermo Fischer Scientific, Braunschweig, Germany, plus 10% α-methyl-cinnamaldehyde). For immunofluorescence, sections were incubated with the corresponding secondary antibody (1:200, donkey anti-rabbit Alexa Fluor 594, ab150064, Abcam, Cambridge, UK, 1:200) for 1 h at room temperature. Cell nuclei were visualized with 4′, 6-diamidino-2-phenylindole (DAPI) and Mowiol was used as a mounting medium. Additional 7 µm-thick paraffin sections were cut from paraffin blocks and mounted on object slides to investigate the presence of hypothalamic di-peptide repeat (DPR) pathology. For this purpose, sections were deparaffinized and treated with 3% H_2_O_2_ in TBS for 20 min. For antigen retrieval, citrate buffer was applied at 100 °C for 20 min. Sections were incubated overnight with the primary mouse antibody against the poly-GA peptide repeat sequence found in C9ORF72/C9RANT (1:2000, Merck Chemicals GmbH, Darmstadt, Germany), followed by incubations with a biotinylated anti-mouse secondary antibody (1:200 for 2 h, Vector Laboratories, Burlingame, CA, USA), and the ABC Vectastain kit (1½ hours, room temperature, Vector Laboratories). The reaction product was visualized with DAB or SK-4700, and the sections were counterstained with Haemalaun or Darrow red, respectively. In negative controls, the absence of the primary antibody incubation resulted in the absence of staining for all markers.

### Histological analyses in human tissue samples

The thick brain sections obtained from paraffin blocks were used for quantifying cell loss in the Pigment Nissl stain and pTDP-43 immunohistochemistry, for visualizing morphological changes in PMCH-positive LHA neurons, and for analyzing the relationship between the TDP-43 pathology and PMCH-positive neurons with immunohistochemistry and immunofluorescence. The severity of pTDP-43 pathology was determined in an image taken from a 70 µm-thick section of each case using an Eclipse LV100ND microscope that was equipped with a digital DS-Fi3 camera and the NIS-Elements software (NIKON GmbH, Düsseldorf, Germany). Image stacks consisting of 40 equidistant images (corresponding to approximately 25 µm section thickness) were captured in the z-axis with the 20X objective from a randomly selected LHA area with a size of 346 µm × 486 µm. Single merged minimum intensity projection images were generated from these image stacks and pTDP-43-containing aggregates were manually counted using the ImageJ software (NIH, Bethesda, Maryland, MD, USA). Cell loss in the LHA was studied using an upright AX10 microscope for bright field and fluorescence microscopy (Zeiss, Jena, Germany) equipped with a Jenoptik Progres Gryphax^®^ Prokyon camera (Jena, Thüringen, Germany). General cell loss was assessed in a 70 µm-thick section of the LHA stained with the pigment Nissl (PN) stain, which visualizes both intracellular and extracellular lipofuscin granules [79, 80]. For this purpose, first the LHA was marked on the object slides and then 4–5 randomly selected digital microphotographs (each covering LHA area of 444 µm × 278 µm) were taken from the LHA with the 40X objective. Quantitative analyses of extracellular lipofuscin aggregates were performed using the ImageJ software version v1.51 k (NIH, Bethesda, Maryland, MD, USA). After opening images, eight grids with a size of 150,000 pixel^2^ were generated resulting in two rows with four grids. Extracellular lipofuscin granules were counted in the first upper grid and last lower grid by tagging the granules with the multipoint count tool and results were recorded. For quantification of PMCH-immunofluorescent neurons, first an overview image was generated with the 10X objective from a 70 µm-thick section of the LHA and the position of PMCH-positive neurons was documented. Next, lateral hypothalamic PMCH-positive neurons without pTDP-43-positive inclusions were manually counted at higher magnification with the 40X objective. The area of the LHA was quantified in the overview image with the ImageJ software by setting the scale and using the free hand tool and analyze/measure commands. Additional images of the LHA were taken from 70 µm-thick sections for documentation of findings with the Eclipse LV100ND microscope (NIKON GmbH, Düsseldorf, Germany). For visualization of complex dendritic morphologies of neurons, Z-stack images were generated using the minimum intensity projection tool of the ImageJ software. In addition, 7 µm-thick sections were used to investigate the presence of DPR inclusions in LHA neurons. Images taken from thick and thin sections were processed with Adobe Photoshop, version 10.0 to correct color faults with photo-filters and to optimize image brightness, hue, and contrast.

### Experimental ALS mice models

All experiments were performed in strict accordance with the Directive European Union 2010/63/UE and the project was approved by the ethical review committee of the University of Strasbourg and the French Department High Education, Research and Innovation.

Wild-type FVB/N and FVB-Tg(SOD1^G86R^)M1Jwg/J (FVB/N background), *SOD1*^*G93A*^ (C57Bl6/J background) and *B6-FusΔNLS1Ldup/Crl* [55, 57] (C57Bl6/N background) were bred in-house. From weaning age, animals were housed in same-sex sibling groups in an environment with controlled temperature and light/dark cycle (12:12 h) with food and water provided ad libitum. Animals were used from 70 to 90 days old or up to 10 months for *Fus* line. Few weeks prior to the procedure, male mice were separated and single housed to habituate.

After the procedure, animals were kept in the environmental control cabinet (CAB-16) under 25 °C, a light/dark cycle (12:12 h) with 200 lux exposures.

### Quantification of MCH neurons using immunofluorescence

Brains were dissected and post-fixed in paraformaldehyde for 24 h. Tissues were cryoprotected into a phosphate buffer saline (PBS) solution with 30% glucose for 72 h, then submerged in isopentane at – 40 ºC and stored at – 80 ºC. Brain slices (thickness 30 μm) were cut using a CM3050S cryostat (Leica Biosystem, Wetzlar, Germany) and collected in PBS with 0.02% Azide. Sections of the region of interest, the LH, were selected using the Allen Mouse Brain Atlas (Allen Reference Atlas—Mouse Brain. Available from atlas.brain-map.org). A total of five sections per animal were stained against MCH and were anatomically matched with their respective controls. Slices were blocked in PBS + 5% horse serum + 0.5% Triton-X-100 for 1 h at room temperature. Primary antibodies were incubated for 2 h at room temperature MCH (H-070-47, 1:2500, Phoenix Pharmaceuticals, Burlingame, CA, USA), diluted in PBS. Slices were washed thrice in PBS and incubated for 1 h at room temperature with a fluorescent conjugated secondary antibody (711-605-152, 1:1000, Jackson ImmunoResearch), diluted in PBS. Three final washes in PBS were done and slices were mounted in VectaShield Plus with DAPI (H-2000-10, Vector Laboratories) on a microscope slide. Imaging was performed on an Axio Imager M2 epifluorescence microscope (Zeiss Laboratories, Thornwood, NY, USA). Tiles were stitched into a 2D plan for further quantification using Zen Pro software. All quantifications were performed by an operator blinded to the genotype or disease status.

### RNAscope fluorescence in situ hybridization

In situ hybridization was performed on 20 μm cryotome coronal brain sections − 1.22 mm and − 1.46 mm caudal from Bregma using RNAscope^®^ Probe- Mm-Pmch-C2.

(Cat No. 478721-C2). Selected sections were mounted on superfrost slides and processed using reported method by manufacturer (Advanced Cell Diagnostics, Newark, CA, ACD, protocol 320,293-USM). Epifluorescent images 20X tile and Z-stack with 1 μm thick optical section were acquired with a Keyence BZ-X800 series microscope, and run by the BZ-X800 analyser software. *Pmch-*positive neurons containing DAPI positive nucleus were manually counted, on maximal intensity projection (MIP) images. Experimenters were blinded to the genotypes performed the neuronal counts.

### Intracerebroventricular injections (i.c.v.)

During the procedure, animals were maintained under deep anesthesia via inhalation of isoflurane (Baxter). The level of anesthesia was verified by testing paw withdrawal reflexes regularly during the procedure. A single injection of meloxicam (Metacam, 2.5 mg/mL; Boehringer Ingelheim) was delivered subcutaneously to the animal during the procedure. The fur was shaved, and the skin was cleaned thoroughly with soapy water and a 70% alcohol solution. A single local subcutaneous injection of lidocaine (Lurocaine, 20 mg, Vetoquinol) was applied under the skin of the skull. The animals were placed in a stereotaxic frame (World Precision Instruments). A small hole was drilled in the skull to permit the insertion of the Brain Infusion Kit in the right lateral ventricle at the following stereotaxic coordinates: bregma − 0.8 mm; midline 0.4 mm; dorsal surface − 2 mm (two adjustment spacers of the Brain Infusion Kit). The Brain Infusion Kit 3 was attached to the Alzet Osmotic Pump (Cupertino, USA) loaded with either the vehicle (saline solution at 0.9% NaCl) or MCH (Tocris #3806, at 0.1 mg diluted in the vehicle solution) and precharged by bathing for approximately 24 h at 37 °C in the vehicle solution prior the procedure. The pump was inserted subcutaneously above the scapulae. The skin was stuck with N-butyl-cyanoacrylate (Surgibond), and the closure was reinforced by a single clip between the scapulae. Finally, animals received a single subcutaneous injection of buprenorphine (Buprecare, 0.1 mg/kg, Axience) to prevent postoperative pain. In addition, 48 h of meloxicam (Metacam, 2 mg/kg) treatment was provided in the drinking water. During surgeries, the experimenter was blinded for the treatment. This was only revealed at the end for statistical analysis to avoid any bias.

### Metabolic assessment: determination of oxygen consumption and carbon dioxide production, respiratory exchange ratio, locomotor activity, and food intake using metabolic indirect calorimetry

We used Promethion CORE (Sable Systems International, North Las Vegas, NV, USA) metabolic indirect calorimetry system to measure physiological parameters in mice with i.c.v. injections while they were maintained on standard chow. Animals were subjected to 1 to 5 days of habituation, depending on the schedule. However, the first night of data collection was always discarded. Animals had access to water and food ad libitum (only food was monitored) as well as to a shelter, which were connected to the system through a mass monitor and permitted food intake and body weight monitoring. Locomotor activity was measured using an XY laser-beam array. Indirect calorimetry was measured using a perforated stainless-steel manifold within the home cage and by a gas analyzer (CGF) situated next to the environmental control cabinet (CAB-16). A pull-mode negative pressure was set to an incurrent flow rate of 2 L/min, and oxygen consumption (VO_2_) and carbon dioxide production (VCO_2_) were captured for each cage every 5 min for 20 s. Reference values from the ambient air were measured every eight cages. Respiratory exchange ratio (RER) was calculated as the quotient of VCO_2_/VO_2_, representing the source of nutrient mobilization (a value of 1 corresponds to 100% of carbohydrate oxidation and 0.7 to 100% of fat oxidation [29]). Energy expenditure (EE) was calculated using the Weir equation [75]. Data were retrieved from the IM3-Interface server and extracted using Sable Systems Macro Interpreter v2.32 with One-Click Macro v2.35 (sliced every 5 min). Eight animals were recorded in parallel, balancing genotype and treatment groups.

### RNA extraction and quantitative reverse transcription-polymerase chain reaction

Total RNA was extracted from snap-frozen tissues using TRIzol® reagent (Sigma). 1 µg of RNA was reverse transcribed with iScript™ reverse transcription (Biorad, 1708841). A quantitative polymerase chain reaction was performed using Sso Advanced Universal SYBR Green Supermix (Bio-Rad) and quantified with the Bio-Rad software. Gene expression was normalized by calculating a normalization factor using the housekeeping genes beta-actin *(Actb)*, TATA box binding protein (*Tbp)*, and *Polr2a* with the GeNorm software [72]. Primer sequences used were as in [73].

### Quantification and statistical analysis

Quantitative parameters obtained for the human brain study were analyzed with the software IBM SPSS Statistics Version 29.0. For comparison of counts of extracellular lipofuscin granules and pTDP-43 aggregates between ALS and control cases, the Mann–Whitney-*U* and Spearman rank correlation tests were used. Group differences in the density of PMCH-positive neurons were analyzed with the aid of the Mann–Whitney-*U* test to compare ALS cases with controls. All data were plotted with the software GraphPad Prism Version 7.0 and presented as mean ± S.E.M. in the graphs. The outcome of statistical analyses was deemed significant at a two-tailed level of *P* < 0.05.

Indirect calorimetric data are shown as plots for the last 56 h of recording. This period corresponded to approximately day 8 to 10 post-pump implantation, when the maximum effect of MCH was observed on body weight in transgenic mice.

For metabolic analysis, we used a generalized linear model ANCOVA analysis with body weight as a covariant for the continuous recordings. Overall metabolism was analyzed using parametric analysis one-way ANOVA followed by Tukey’s multiple comparisons test. Because of the allometric relationship between body weight and metabolism, the data were not normalized to the body weight [67, 71]. Experiments were analyzed using the software CalR version 1.3 [47].

## Results

### Loss of MCH-expressing neurons in three mouse models of ALS

**Fig. 1:**
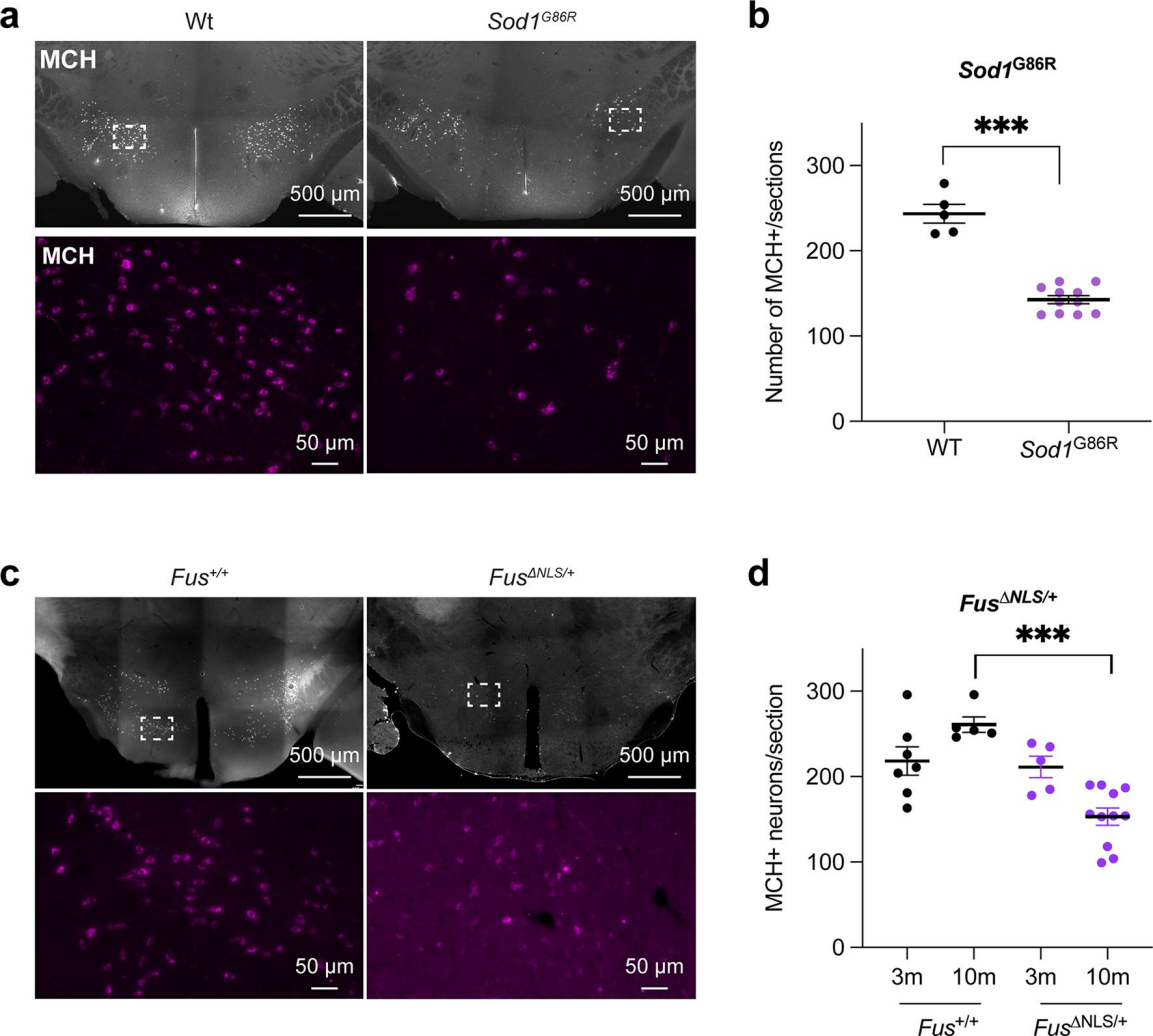
Loss of MCH-positive neurons in ALS mouse models. a: Representative MCH immunostaining in *Sod1*^G86R^ or wild-type littermates at 90 days of age (prior to motor symptom onset). The lower panels show higher magnification of the region of interest indicated by the dashed rectangle. b: Number of MCH-positive cells per sections in *Sod1*^G86R^ (*n* = 11) or wild-type (WT, *n* = 5) littermates at 90 days of age (prior to motor symptom onset).. *** *P* value < 0.0001, Unpaired *t *test. Data are presented as mean and S.E.M. c: Representative MCH immunostaining in 10-month old *Fus*^*∆NLS/*+^ or *Fus*^+*/*+^ littermates. The lower panels show higher magnification of the region of interest indicated by the dashed rectangle. d: Number of MCH-positive cells per sections in 3-month old *Fus*^*∆NLS/*+^ (*n* = 5) or *Fus*^+*/*+^ (*n* = 7) littermates and in 10-month old *Fus*^*∆NLS/*+^ (*n* = 11) or *Fus*^+*/*+^ (*n* = 5) littermates. *** *P* value < 0.0001, Unpaired *t* test. Data are presented as mean and S.E.M.

Since previous evidence showed alterations in the LHA in ALS, we studied different mouse models of ALS and counted the number of MCH-positive neurons in the region (Fig. 1). We used the *Sod1*^G86R^ (Fig. 1a, b) and *SOD1*^*G93A*^ (Supplementary Fig. 1) mouse lines that express different mutations of SOD1 linked to ALS. We observed a significant decrease in the number of MCH-positive neurons (Fig. 1b**)**. Similarly, in *Fus*^∆NLS/+^ mice, a conditional knock-in mice expressing an ALS-like truncated FUS protein [60, 63], MCH neuronal positive cells were similar to wild-type littermate mice at 3 months of age yet decreased at 10 months of age, an age at which cognitive and motor symptoms are present [61, 62] (Fig. 1c, d).

### Chronic i.c.v. MCH supplementation rescues weight deficit and increased food intake in male Sod1^G86R^ mice

**Fig. 2:**
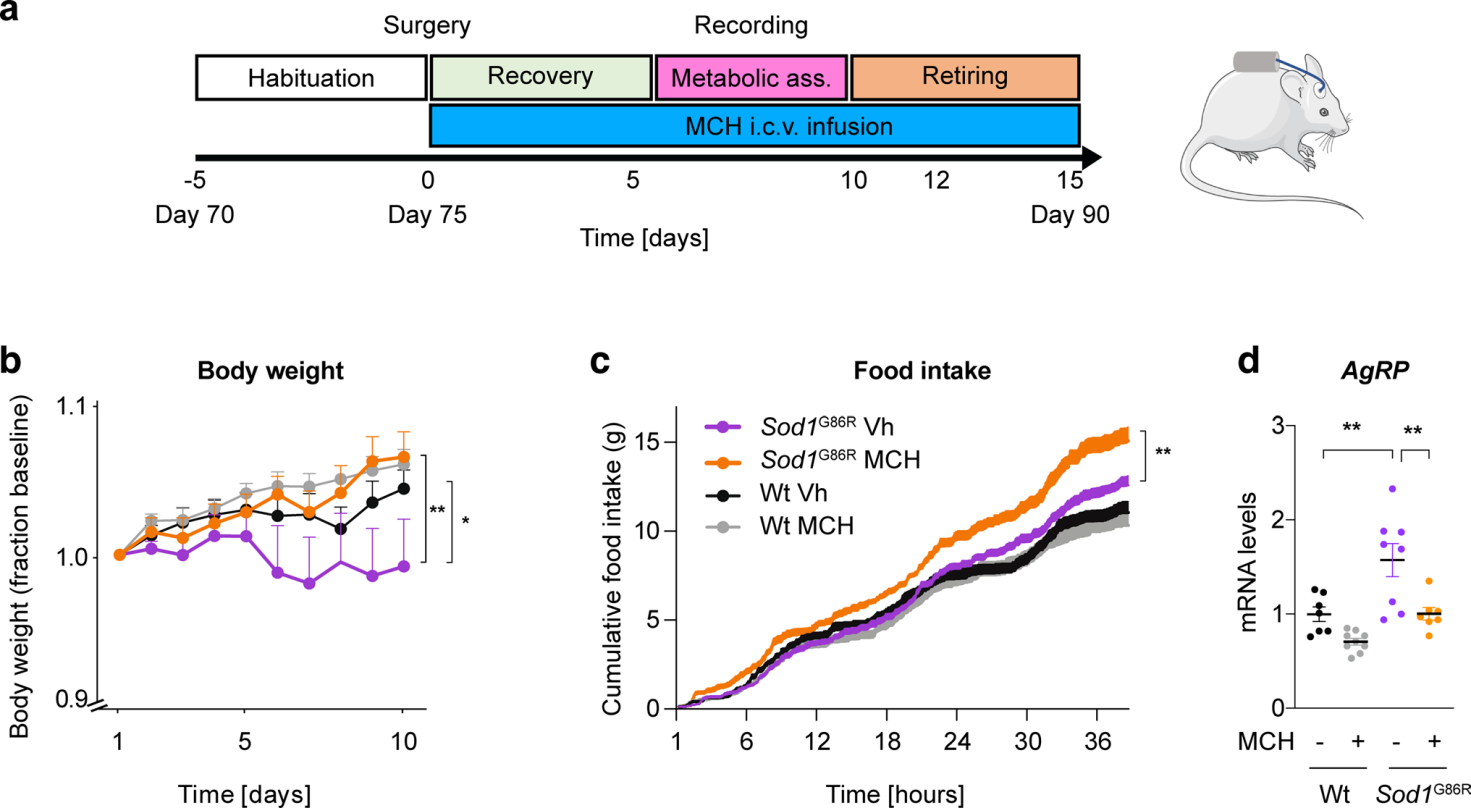
MCH i.c.v. delivery rescues weight loss in male *Sod1*^*G86R*^ mice through increased food intake. **a**: Experimental design; **b**: changes in body weight ratios over the 15 days of the MCH delivery (*n* = 8 per group). ANOVA one-way test (*P* value < 0.0001) followed by Tukey’s *post hoc* analysis Wt Vh vs *Sod1*^G86R^ Vh * (*P* value = 0.003) and *Sod1*^G86R^ MCH vs *Sod1*^G86R^ Vh ** (*P* value = 0.0002); **c**: cumulative food intake in male mice over the 15 days of the MCH delivery (n = 8 per group). ANOVA two-ways test (*P* value < 0.004) followed by Dunnett’s *post hoc* analysis *Sod1*^G86R^ MCH vs *Sod1*^G86R^ Vh ** (*P* value = 0.002); **d**: *Agrp* gene expression in hypothalamus of indicated mouse groups. **, *P* value < 0.01 ANOVA followed by Tukey’s multiple comparison test

We asked whether the loss of MCH could account for the weight deficit observed in *Sod1*^G86R^ mice and whether its central supplementation could delay it. We chose this model because of their rapid and severe disease course [57], well-documented metabolic defects [24, 54], and lack of confounding effects of SOD1 overexpression on mitochondrial metabolism as seen in *SOD1*^G93A^ mice [4, 36, 37]. We, thus, used 75 day-old *Sod1*^G86R^ mice and their control littermates to implant an osmotic mini-pump under the skin linked to an i.c.v. cannula in the lateral ventricle (Fig. 2a). Pumps were filled with either the vehicle or 0.1 mg of MCH. This allowed a constant delivery of the hormone at a rate of approximately 1.2 µg/day for 15 days. In these experiments, implanted mice were allowed to recover for 5 days. This was followed by 4 days of metabolic assessment recorded by indirect calorimetry (Fig. 2a). Remarkably, the dose used was three times lower than the already reported doses known to increase food intake in wild-type mice [35]. The i.c.v. administration of 0.1 mg MCH led to weight gain in *Sod1*^G86R^ male mice but not in wild-type controls (Fig. 2b). The effect on weight deficit was associated with an increased cumulative food intake in *Sod1*^G86R^, but not in wild-type mice (Fig. 2b). We previously showed that *AgRP* expression was increased in *Sod1*^G86R^ mice, like in fasted wild-type mice [73]. MCH supplementation in *Sod1*^G86R^ mice led to *AgRP* mRNA levels similar to wild-type untreated mice (Fig. 2d). Thus, MCH supplementation was sufficient to change hypothalamic gene expression, increase food intake, and maintain a normal body weight in *Sod1*^G86R^ mice.

### Metabolic effects of chronic i.c.v. MCH supplementation in Sod1^G86R^ mice

**Fig. 3:**
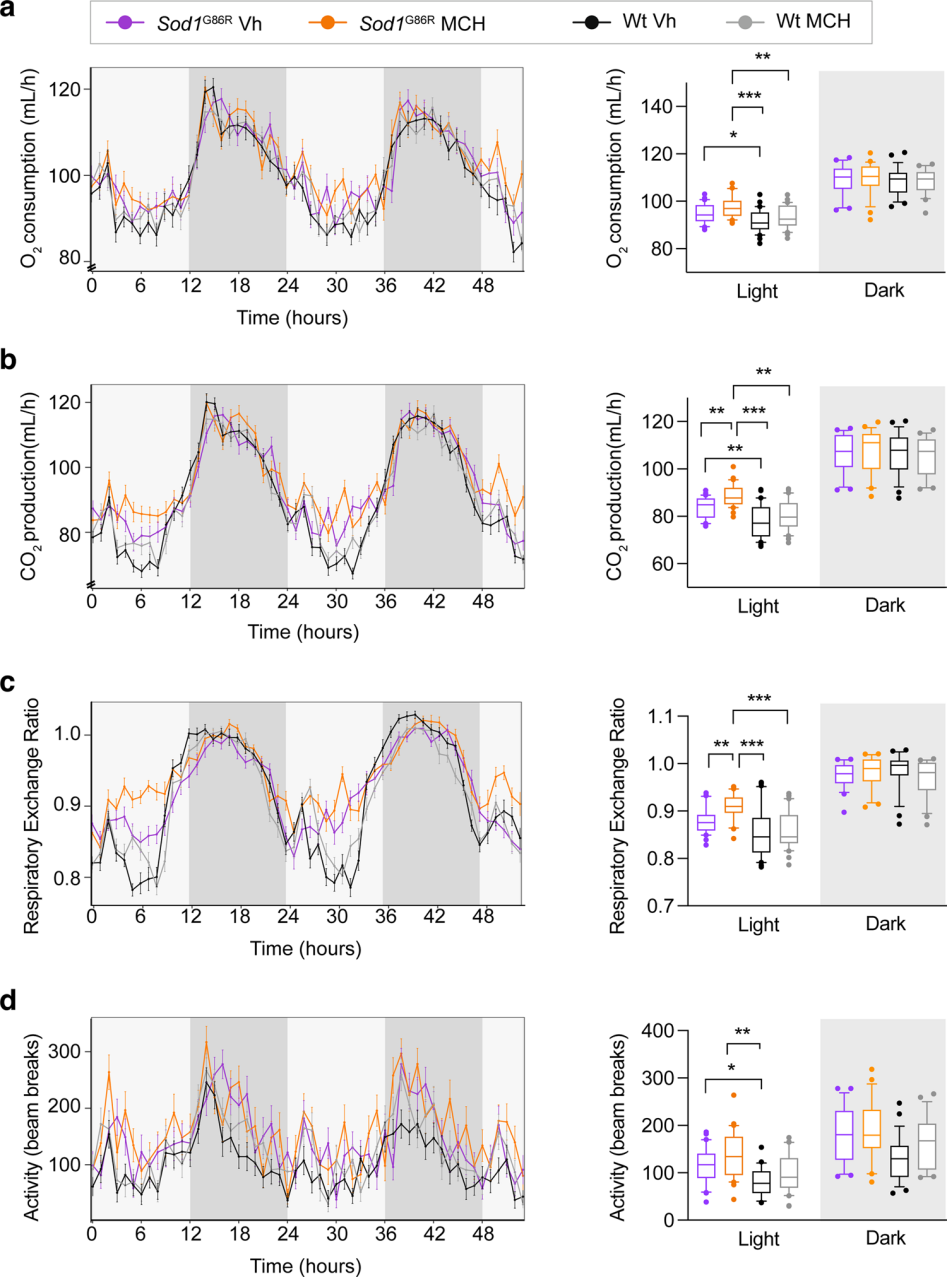
Effect on metabolism after MCH i.c.v. injection in male *Sod1*^*G86R*^ mice. **a–d**: Mean traces of O_2_ consumption (**a**), CO_2_ release (**b**), respiratory exchange ratio (RER) (**c**), and locomotor activity (**d**) in male *Sod1*^G86R^ mice treated with vehicle (purple) or MCH (orange) and in wild-type littermates treated with vehicle (black) or MCH (grey). The generalized linear model analysis using specifically the ANCOVA analysis was used (with body weight as a covariate). CalR interface was used to generate the analysis [47] The left panels show two consecutive days of recording, while the right panels show boxes and whiskers plots for the same groups in the light and dark periods. Note the effect of MCH on increased respiratory exchange ratio in the light phase in *Sod1*^G86R^ mice. *, *P* value < 0.05; ***, *P* value < 0.001 one-way ANOVA with *post hoc* Tukey’s multiple analysis

We then analyzed the effects of MCH supplementation on energy metabolism. Mice are nocturnal animals, and their energy metabolism is different between day and night. During the day, (*i.e.* “light phase”), animals are inactive, sleep, and their metabolism is at rest. At night, (*i.e.* “dark phase”), mice are active and increase their metabolism. To determine whether MCH had general effects on metabolism, we used a generalized linear model ANCOVA analysis over the whole period. This analysis showed no effect of MCH on energy expenditure (Fig. 3a), CO_2_ release (Fig. 3b), RER (Fig. 3c), and locomotor activity (Fig. 3d) in males. However, when light and dark phases were separated for the analysis (right panels of Fig. 3), we observed an increase in CO_2_ release (Fig. 3b) and RER specifically during the light phase in *Sod1*^G86R^ mice after MCH treatment (Fig. 3c). This suggested an increased usage of carbohydrates during the inactive period.

**Fig. 4:**
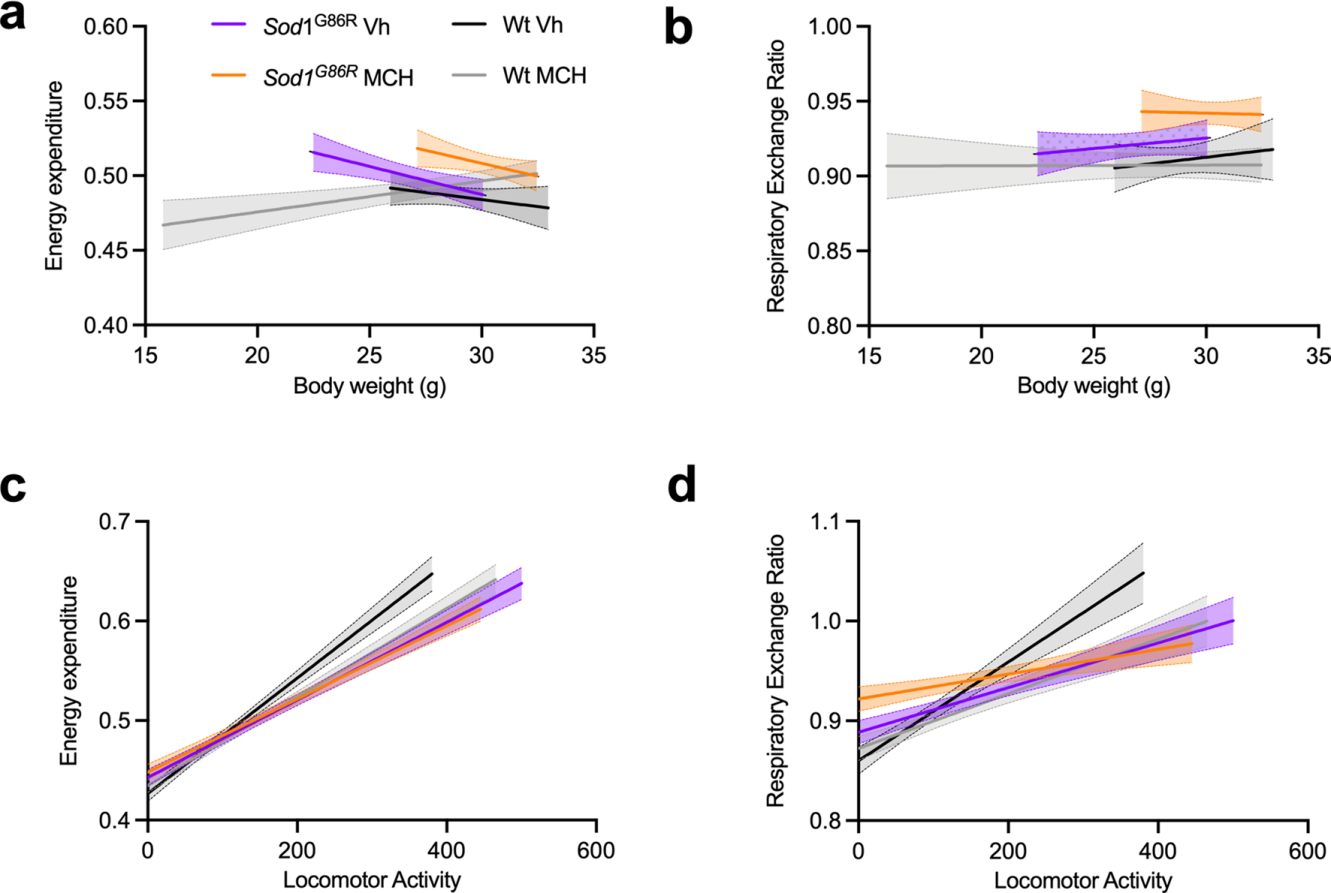
Correlation analysis of MCH i.c.v. injection in male *Sod1*^*G86R*^ mice **a**–**b**: correlation between energy expenditure (**a**) or respiratory exchange ratio (**b**) and body weight in male *Sod1*^G86R^ mice treated with vehicle (purple) or MCH (orange) and in wild-type littermates treated with treated with vehicle (black) or MCH (grey). Note that MCH injection increases energy expenditure and RER relative to body weight. **c**–**d**: correlation between energy expenditure (**c**) or respiratory exchange ratio (**d**) and locomotor activity in male *Sod1*^G86R^ mice treated with vehicle (purple) or MCH (orange) and in wild-type littermates treated with vehicle (black) or MCH (grey). MCH injection increases energy expenditure and RER relative to locomotor activity in wild-type but not in *Sod1*^G86R^ mice

To better visualize the differences, we correlated energy expenditure and RER with body weight and locomotor activity, respectively (Fig. 4). This analysis revealed that *Sod1*^G86R^ mice had increased energy expenditure and RER relative to their body weight, which means that, at similar body weight, they show increased energy expenditure compared to wild-type mice. This is equivalent to a “hypermetabolism”-like state (Fig. 4a, b) and has been described in multiple previous studies [24, 39, 58, 59, 69]. Interestingly, MCH further increased energy expenditure in *Sod1*^G86R^ mice, showing that the rescue in body weight was not due to decreased “hypermetabolism” in *Sod1*^G86R^ mice. Interestingly, MCH also increased RER in relation to body weight (Fig. 4b). An increasein RER demonstrates an augmentation usage of carbohydrate (RER = 1) and a decrease an usage predominantly of lipids (RER = 0.7). Here, we showed that MCH increases carbohydrate use in *Sod1*^G86R^ mice. This was most noticeable at low activity rates as treated *Sod1*^G86R^ mice showed elevated RER when they were less active (Fig. 4c-d). Overall MCH supplementation in *Sod1*^G86R^ mice does not rescue “hypermetabolism” but appears to induce a shift in fuel usage related to activity.

### Cell loss in the lateral hypothalamus in human sporadic ALS

**Fig. 5:**
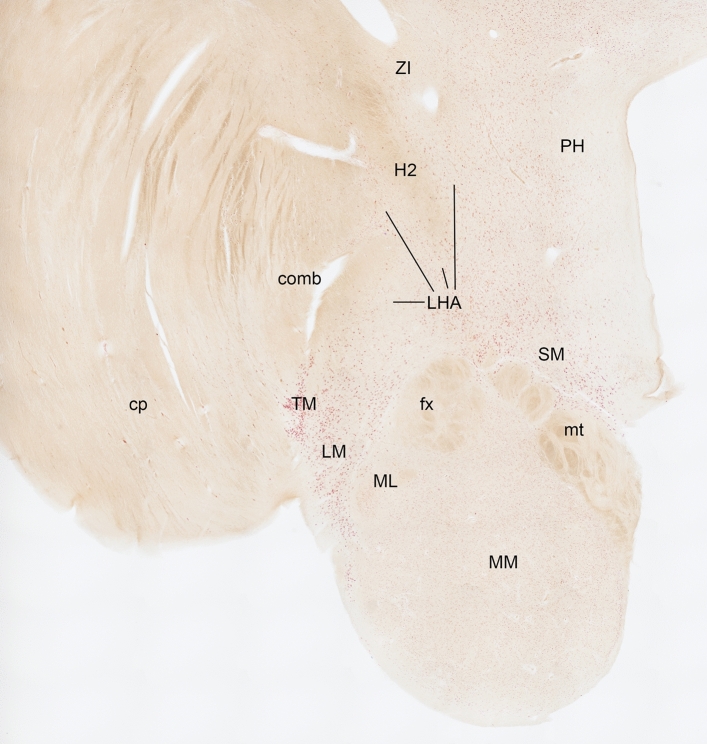
Overview of the human hypothalamus of a control case stained with the pigment Nissl (PN) stain. Delineation of the LHA in the human hypothalamus for quantification of extracellular lipofuscin granules in the PN stain. Upon zooming into the image, different neuronal populations with intracellular lipofuscin pigment can be distinguished in the LHA. The stitched image of the hypothalamus was taken at high resolution (4X objective, Eclipse LV100ND Nikon microscope) and downsized to 20% of the original image size. *Abbreviations:* *comb* comb system, *cp* cerebral peduncle, *fx* fornix, *H2* lenticular fasciculus (H2-field), *ML* medial mammillary nucleus, lateral part, *MM* medial mammillary nucleus, medial part, *mt* mammillo-thalamic tract, LHA lateral hypothalamic area, LM lateral mammillary nucleus; *PHA* posterior hypothalamic area, *SM* supramammillary nucleus, *TM* tuberomammillary nucleus, *ZI* zona incerta

**Fig. 6:**
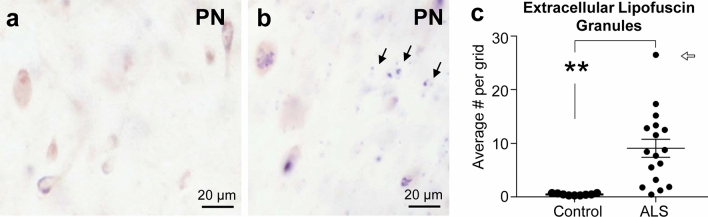
Extracellular lipofuscin granules in postmortem LHA tissue of ALS patients and controls. **a**: Neurons in the LHA of a control case with a red cytoplasm (labeled with Nissl staining using Darrow red) and blue intracellular lipofuscin granules (stained with aldehyde fuchsine) as seen in the pigment Nissl (PN) stain. **b**: Neurons and extracellular lipofuscin pigment granules (black arrows) in the LHA of an ALS patient. **c**: Counts of extracellular lipofuscin pigment granules performed by blind observer in postmortem LHA tissue of ALS patients compared to control patient tissue. Open arrow points to Case # 10. **c**. ** *P* value < 0.01, Mann–Whitney test. Data are presented as mean and S.E.M

We then asked whether the observed LHA deficits could also be observed in sporadic ALS patients and determined the presence of neuronal damage in the LHA of ALS patients in sections marked with Nissl staining. Figure 5 shows an overview of the human hypothalamus stained with pigment Nissl (PN). We then counted extracellular lipofuscin granules that form disorganized aggregates outside the cytoplasm of Darrow red-stained neurons (Fig. 6). These are remnants of neurons left behind after neuronal cell death as seen in several neurological diseases (e.g., [10, 79, 80]). We observed a significantly higher amount of extracellular lipofuscin granules in the LHA of ALS cases compared to controls (ALS: *n* = 17, controls: *n* = 9, Mann–Whitney test, *U* value = 5, exact two-tailed *P* value < 0.0001). This supports the presence of substantial lateral hypothalamic neuronal cell damage in ALS.

### pTDP-43 pathology in the lateral hypothalamus in ALS patients

**Fig. 7:**
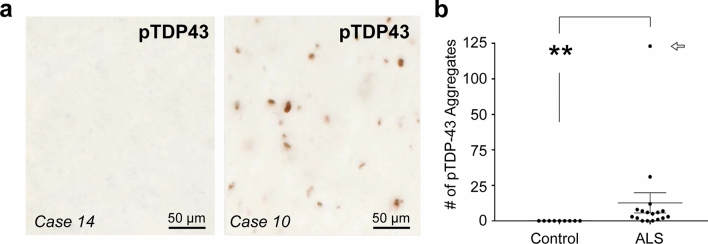
pTDP-43-immunoreactive aggregates studied in postmortem tissue of ALS patients and of controls. **a**: Representative images of the LHA showing an ALS case lacking pTDP-43-positive pathology in the LHA (Case 14) and another ALS case with severe pTDP-43 pathology (Case 10) in this brain region (scale bar 50 µm). **b**: Counts of pTDP-43 aggregates in the LHA obtained in postmortem tissue of ALS patients compared to control cases. Open arrow points to count of case # 10 (b). ** *P* value (exact sig.) < 0.01, Mann–Whitney test. Data are presented as mean ± SEM

We characterized the extent of phosphorylated TDP-43 (pTDP-43) pathology, which is the major component of ubiquitin-positive inclusions in ALS [13, 50], in the LHA of patients and controls (Fig. 7). Aggregates of pTDP-43 were observed in the LHA of 100% of cases with ALS stage 4 (seven out of seven cases), 75% of cases with ALS stage 3 (three out of four cases), 100% of cases with ALS stage 2 (six out of six cases), and 0% of cases with ALS stage 1 (one case only). Quantitative analyses revealed a significant difference between the pTDP-43 pathology in the LHA of ALS cases and controls (ALS: *n* = 17, controls: *n* = 9, Mann–Whitney test, *U* value = 13.5, exact two-tailed *P* value < 0.01, difference = 5). Moreover, the density of pTDP43 aggregates in the LHA significantly correlated with the density of extracellular lipofuscin granules (Spearman rank correlation, *r* = 0.639, *P* < 0.001, *n* = 26). Indeed, the ALS case (Case # 10), which had the highest (and a particularly high) density of pTDP-43-positive aggregates in the LHA (Fig. 7b), also had the highest density of extracellular lipofuscin granules in this brain region (Fig. 6c).

### pTDP-43 inclusions in lateral hypothalamic PMCH neurons

**Fig. 8:**
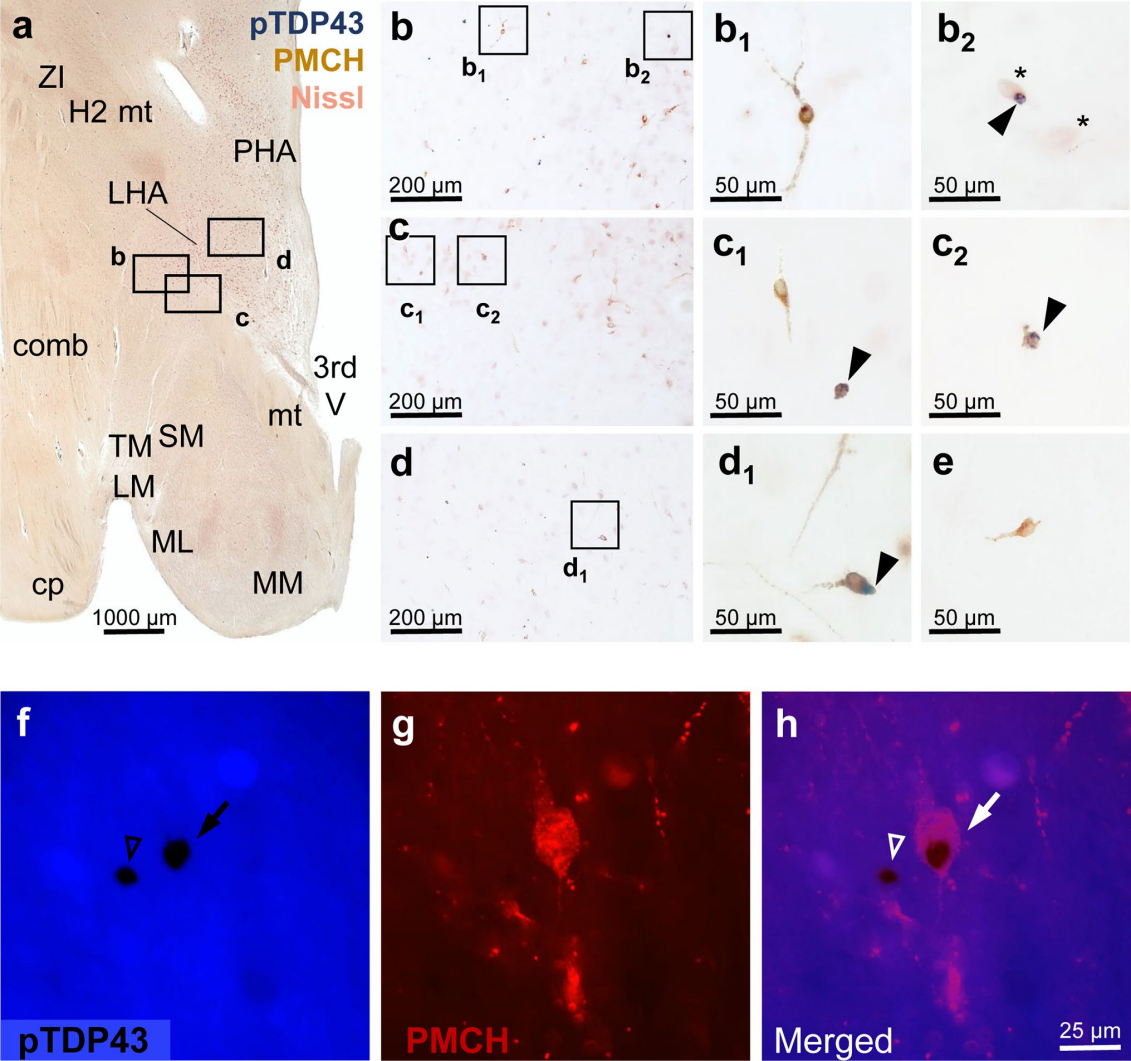
pTDP-43-immunoreactive cytoplasmic inclusions in PMCH expressing neurons in the LHA of ALS patients. Double-label immunohistochemistry (**a**–**e**) and double-labeling performed by combining immunohistochemistry with immunofluorescence (**f**–**h**) show PMCH neurons in the LHA with pTDP-43-positive inclusions. *Upper tier*—**a**: Overview of the human hypothalamus of ALS patient studied using double-label immunohistochemistry showing pTDP-43-immunoreactive intracellular inclusions (blue) and PMCH-positive neurons (brown) counterstained with Darrow red (Nissl staining). **b**, **c,** and **d**: Mid-level magnification of insets from (**a**) that show the position of individual neurons studied at higher magnification with new insets (insets b1-d1). *Inset d1:* PMCH neuron with pTDP-43-positive inclusion (arrow). *Inset b2:* Among the two non-PMCH neurons shown (stars), one non-PMCH neuron contains a pTDP-43-positive inclusion (arrow). *Insets c1 and c2:* Glial cells with pTDP-43-positive inclusions (arrow). *Insets b1, c1*,* and e:* PMCH neurons without pTDP-43-positive inclusions. *Lower tier—***f**–**h**: DAB-labeled pTDP-43-positive inclusion (black) captured in blue channel (**f**), PMCH-immunofluorescent neuron (**g**), and merged image (**h**) in the human LHA. *Abbreviations:* *3rd V* third ventricle, *comb* comb system, *cp* cerebral peduncle, *H2* lenticular fasciculus (H2-field), *ML* medial mammillary nucleus, lateral part, MM medial mammillary nucleus, medial part, *mt* mammillo-thalamic tract; *LHA* lateral hypothalamic area, *LM* lateral mammillary nucleus, *PHA* posterior hypothalamic area, *SM* supramammillary nucleus, *TM* tuberomammillary nucleus, *ZI* zona incerta

**Fig. 9:**
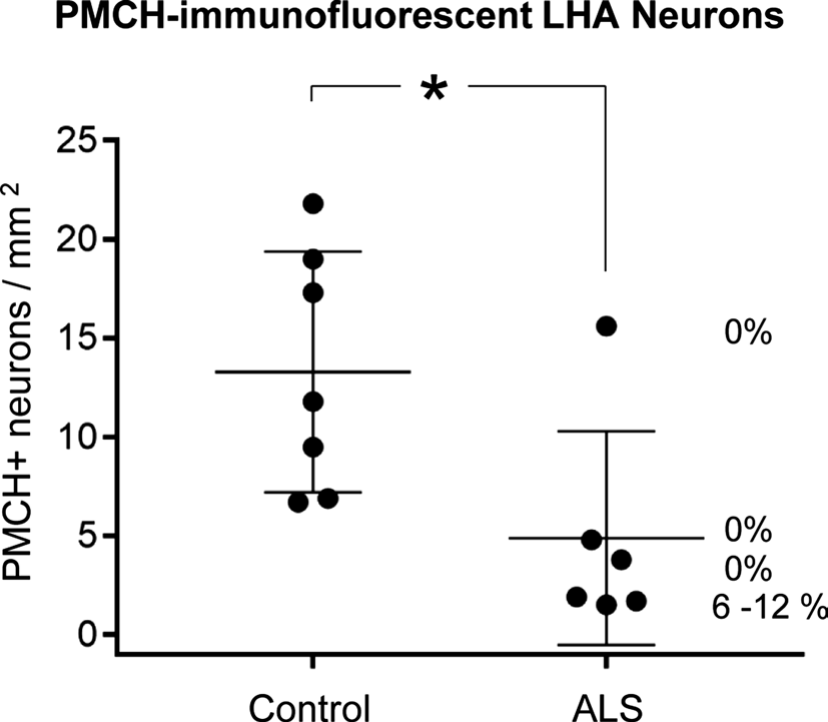
Quantification of the density of PMCH-immunofluorescent neurons and of the percentage of PMCH-positive neurons with pTDP-43 inclusions in the LHA of ALS patients compared to controls. The density of PMCH-positive neurons was significantly reduced in ALS cases compared to controls. The LHA of all ALS patients analyzed here exhibited pTDP-43 pathology, but only a subset of these ALS patients had pTDP-43-containing inclusions in PMCH-positive neurons. The percentage of PMCH-positive neurons with pTDP-43 inclusions is shown next to the value of each ALS case. The density of PMCH-positive neurons in the LHA was particularly low in ALS cases with pTDP-43-positive inclusions, which were found in 6–12% of PMCH-positive neurons. Mann–Whitney-*U* test, *P value < 0.05. Data are presented as mean and S.E.M.

To study pTDP-43 proteinopathy in PMCH-positive neurons in ALS, we performed double-label immunohistochemistry and immunofluorescence. Our results show pTDP-43-immunoreactive inclusions in LHA neurons-expressing PMCH (Fig. 8), although non-PMCH neurons and glial cells also showed pTDP-43-positive aggregates (Fig. 8). While the percentage of PMCH-positive neurons with pTDP-43 inclusions in the LHA of ALS patients was variable, altogether the ALS cases had a significantly lower density of lateral hypothalamic PMCH-positive neurons than the controls (Fig. 9) as indicated by statistical analyses (Mann–Whitney test, *U* value = 38, *n* = 13, exact two-tailed *P* value = 0.014). The density of PMCH-positive neurons in the LHA was particularly low in ALS cases, in which PMCH-positive neurons contained pTDP-43-positive inclusions (in 6–12% of PMCH-positive neurons; see Fig. 9).

### Atrophy and neurodegeneration in PMCH neurons of the LHA in human ALS

**Fig. 10:**
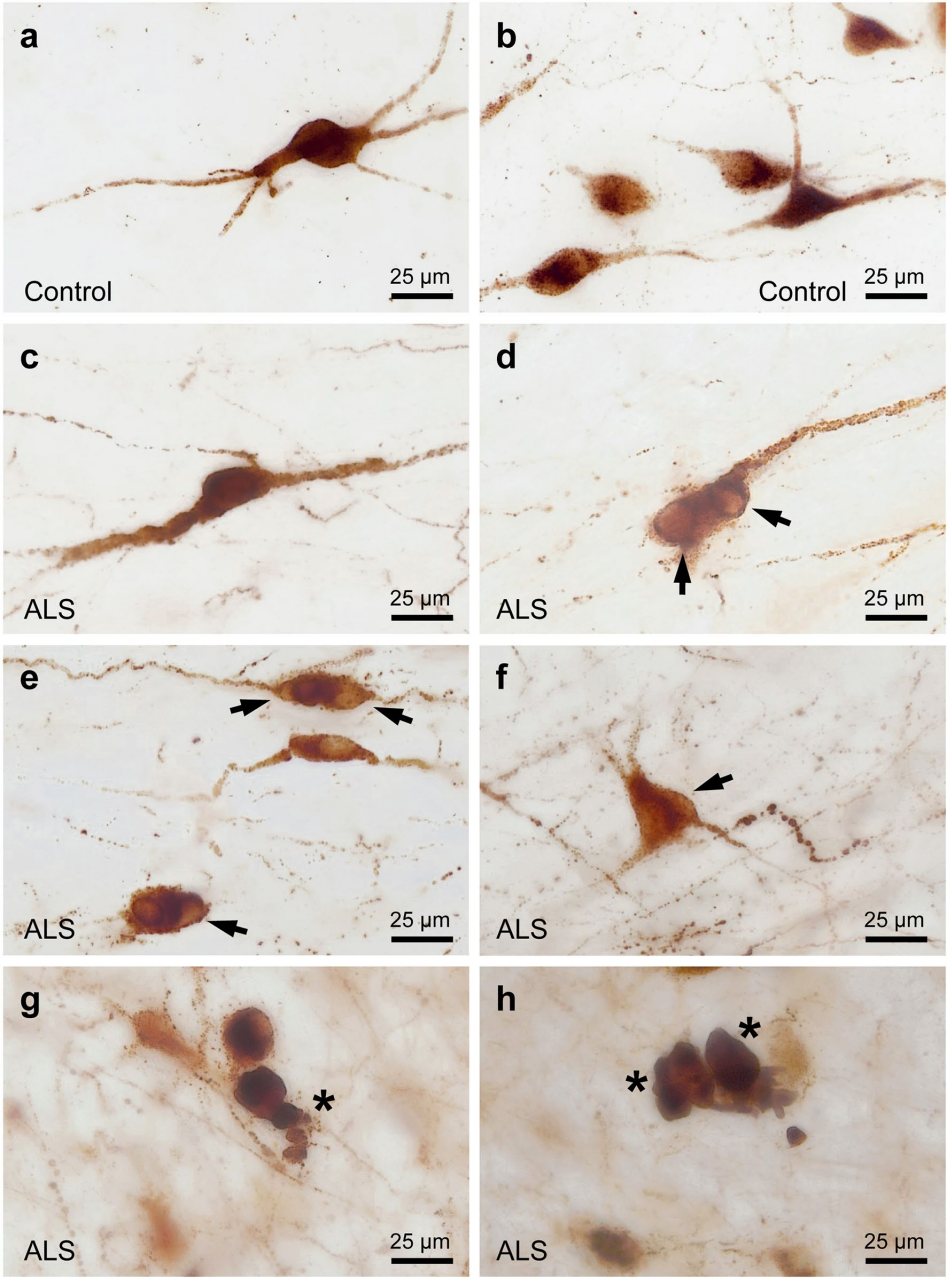
Degenerating PMCH-immunoreactive neurons in the LHA in human ALS. **a**–**b**: Morphology of PMCH-positive neurons in control cases. **c**: PMCH-positive neurons in an ALS case without morphological abnormalities. **d**–**f**: Examples of PMCH-positive neurons with inclusion bodies resulting in protrusions of the cytoplasm (arrows). **g**–**h**: Degenerating PMCH-labeled neurons with hyaline-appearing cytoplasm, irregular somata, and lack of dendritic processes (stars). This pathology was preferentially found in the LHA of ALS cases with a rather low or very low density of PMCH-positive neurons

**Fig. 11:**
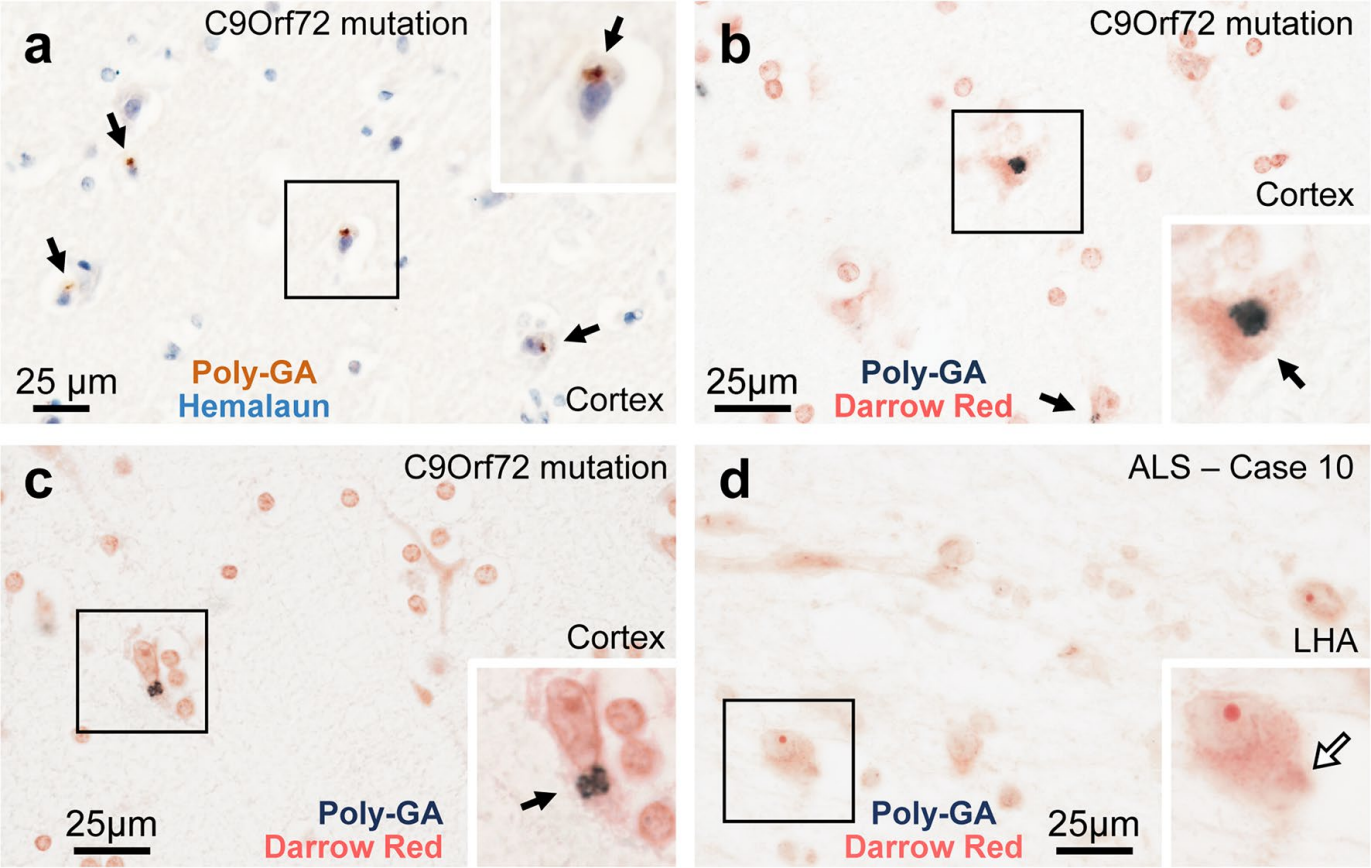
Analysis of poly-GA-containing di-peptide repeat (DPR) aggregates in the human hypothalamus studied using immunohistochemistry. **a**–**c**: Intraneuronal poly-GA-containing DPR aggregates in the cortex of a positive control case with clinically diagnosed C9Orf72 hexanucleotide repeat expansion. **d**: Examples for neurons in the LHA of our ALS cases, which were devoid of intraneuronal poly-GA-containing DPR aggregates (here in ALS case # 10 with the highest extracellular lipofuscin granule and pTDP-43 aggregate counts). The poly-GA aggregates were visualized with the brown reaction product of DAB (**a**) or with the dark blue reaction product of SK4700 (**b**–**d**), counterstained with Haemalaun and Darrow red, respectively. Boxed areas in the images are shown at higher magnification in the insets. Black arrows: poly-GA-containing DPR aggregates. Open arrow: intraneuronal inclusion that is poly-GA-negative. Scale bars: 25 µm

As indicated above, some ALS patients with pTDP-43 inclusions in PMCH neurons showed particularly low densities of PMCH neurons (Fig. 9). In these ALS cases, we detected individual PMCH-positive neurons with signs of neuronal atrophy and neurodegeneration, further supporting loss of PMCH neurons in ALS. Such degenerative changes in PMCH neurons included protrusions of the cytoplasm with PMCH-negative inclusion bodies (Fig. 10), which morphologically resembled the protruding pTDP-43-positive inclusions seen in double-labeling for PMCH and pTDP-43 (see Fig. 8 for comparison). Additional neuropathological analyses in the LHA of our ALS cohort failed to show presence of intracellular DPR aggregates (Fig. 11), which were studied as another potential likely source of proteinopathy in inclusions of PMCH-positive neurons, since approx. 7% of sporadic ALS cases have C9Orf72 hexanucleotide repeat expansions known to cause DPR aggregation [45]. Occasionally, hyaline cytoplasmic PMCH labeling with fragmented neuronal somata and dendrites was detected in the LHA of ALS cases, which had pTDP-43 inclusions and particularly low densities of PMCH-positive neurons (Fig. 10).

## Discussion

This study demonstrates that MCH neurons are affected in ALS, both in patients and animal models. Central complementation of MCH rescues weight loss in male mutant SOD1 mice through increased food intake and modifies energy metabolism as a function of locomotor activity. These two results have consequences for our understanding of disease pathogenesis and provide avenues to pharmacologically target weight loss in ALS.

Our first important result is that MCH neurons are affected in the LHA in both ALS patients and ALS mouse models. Previous research has observed pTDP-43 pathology in the hypothalamus [14, 15, 31, 49]. Similar to Cykowski and collaborators [14], we confirm here the occurrence of pTDP-43 pathology in the LHA of ALS patients. The hypothalamic involvement observed in ALS aligns with the evidence of hypothalamic atrophy detected through MRI studies by us and other researchers [34, 40] and pathological investigations [31]. Using pigment Nissl staining, we could further show a significant neuronal cell loss in the LHA of ALS patients compared to controls. A reduced hypothalamic volume in general and particularly in the LHA has been linked to a reduced body mass index (BMI), suggesting a role of the hypothalamus in the metabolic dysregulation in ALS patients [14, 34].

Among the different neuronal types in the LHA, we now show that MCH neurons are affected in ALS. In sporadic ALS patients, the density of MCH neurons was reduced compared to controls, and we observed a decrease in the number of MCH-positive neurons in three mouse models of ALS. This finding echoes the observation of Gabery and collaborators [31] who showed loss of neurons-expressing orexin, the other major LHA neuropeptide, in human ALS, which correlated with changes in eating behavior. While we further detected pTDP-43-positive cytoplasmic inclusions in MCH neurons in our sporadic ALS patients, these inclusions were not limited to MCH neurons, but they were also observed in other LHA neuronal types and in glia in our study. Moreover, the density of pTDP-43-positive aggregates significantly correlated with the density of extracellular lipofuscin granules in the LHA, supporting the contribution of TDP-43 pathology to the loss of both MCH neurons and other neuronal populations. Intriguingly, not all ALS cases studied here showed hypothalamic TDP-43 pathology in agreement with previous investigations. Hence, only a subgroup of ALS patients with pTDP-43 pathology in the LHA showed MCH neurons with pTDP-43 inclusions and MCH neurons with rare but conspicuous morphological degenerative features. A possible cause could be how the disease spreads in these patients and affects hypothalamic nuclei [40].

What could be the consequences of MCH loss? MCH is a multi-faceted neuropeptide with various effects on energy homeostasis and physiology [2], including increasing food intake, decreasing energy expenditure, rewiring of metabolic pathways in the liver and adipose tissue, as well as decreasing locomotion. Loss of MCH leads to leanness, which is mostly caused by increased energy expenditure, and is associated with hypophagia in mice [42, 65]. Loss of MCH is also known to affect the melanocortin system [65], and increase energy expenditure in obese mice as well as in aged mice [38, 64], while conversely, acute injection or overexpression of MCH triggers obesity [43], decreases energy expenditure [33] and sympathetic tone [25]. To determine how MCH loss could contribute to ALS-related abnormalities in energy homeostasis, we rescued MCH deprivation by i.c.v. delivery of MCH in a cohort of male *Sod1*^*G86R*^ mice. In these mice, the weight deficit was rescued, and this was associated with increased food intake, although energy expenditure or locomotion remained unchanged. MCH also had subtle effects on other metabolic parameters, but our data are in favor of the notion that the major consequence of MCH loss is inadequate food intake in ALS. Beyond energy metabolism, MCH is also involved in sleep regulation [56], and interestingly, we observed more profound effects of MCH supplementation during the light phase, i.e., the inactive period in mice. Whether sleep defects occur because of hypothalamic loss of MCH neurons in ALS is an open question that deserves further investigation.

A major question arising from our results is how MCH neurons, and more generally the LHA, is linked to ALS progression. According to the centrifugal model of disease pathogenesis by Braak and collaborators [9, 13], pTDP-43 pathology spreads from the motor cortex to anatomically connected regions. MCH neurons are anatomically connected to the sensory-motor cortex [26]. In general, the LHA is densely connected to the cortex as well as to many other brain and spinal cord areas [5, 6]. Hypothalamic atrophy is more severe in stage 3/4 ALS patients in both diffusion tensor imaging-based staging and pathological studies [14, 34]. Moreover, cortico-hypothalamic pathways have been shown to be disrupted in ALS mouse models and patients [3]. It is, thus, conceivable that the involvement of MCH neurons is a secondary consequence of disease progression, contributing to the deficit in energy intake failing to match the energy needs as observed in patients [1, 51, 68]. Hence, MCH receptor agonists may be useful in treating weight loss in ALS patients, which is particularly relevant considering that drugs known to increase food intake through the POMC/AgrP pathway were inefficient in increasing weight in ALS patients [73].

Our study has several limitations. First, we had no *SOD1* or *FUS* autopsy cases that could directly translate our findings in mice to patients carrying similar mutations. We also have no or limited information available on the metabolism (e.g., weight loss, food intake or energy expenditure) of ALS patients studied here that would mirror our experimental studies in mice. However, the commonalities observed in MCH neuronal loss in various models, with different causes, and in a subset of sporadic cases, argues for MCH loss being clinically relevant in ALS disease.

Altogether, our current study identifies MCH neurons as a hypothalamic neuronal cell type, the impairment of which contributes to inadequate food intake in ALS, and which may be a new target for combatting ALS-induced deficits in energy metabolism.

## Supplementary Information

Below is the link to the electronic supplementary material.

Supplementary file1 (PDF 527 KB)

## Data Availability

All data generated or analyzed during this study are included in this published article.
