# Supplementary material for: Loss of hypothalamic MCH decreases food intake in amyotrophic lateral sclerosis
Source: Acta Neuropathol. 2023 Apr 14;145(6):773–91. doi: 10.1007/s00401-023-02569-x (PMC10175407; doi:10.1007/s00401-023-02569-x)
Supplement: Supplementary file 1 — Supplementary file1 (PDF 527 KB) [file 401_2023_2569_MOESM1_ESM.pdf]

Loss of hypothalamic MCH decreases food intake in amyotrophic lateral sclerosis

Bolborea and collaborators.

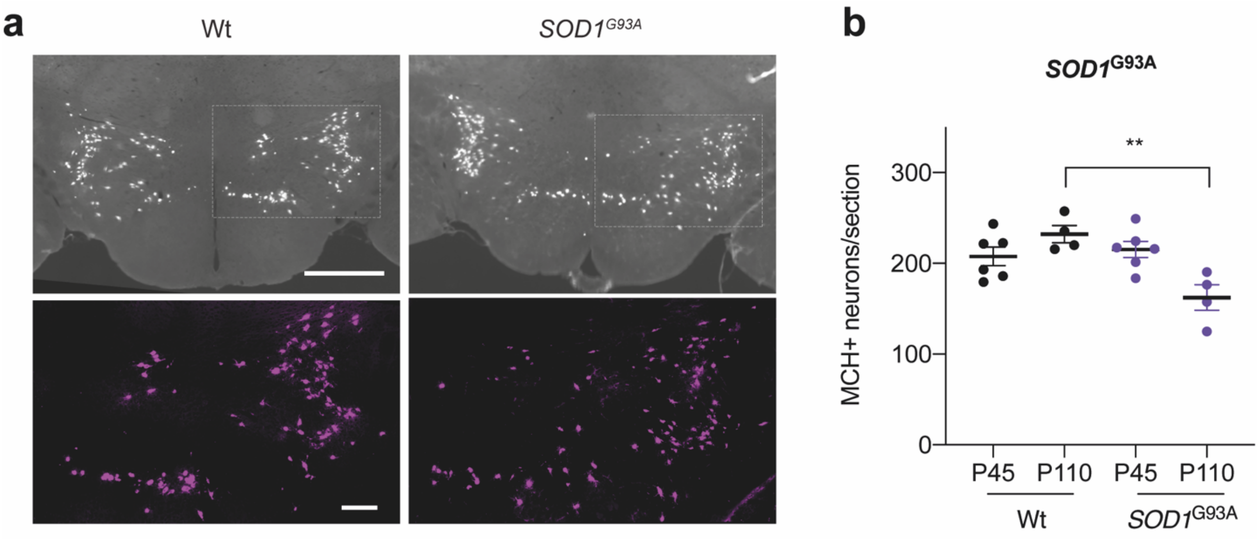

Supplementary Figure 1: Loss of MCH positive neurons in *SOD1*^{G93A} mice.

a: Representative MCH *in situ* hybridization in *SOD1*^{G93A} or wild-type (WT) littermates at 110 days of age (prior to motor symptom onset). The lower panels show higher magnification of the region of interest indicated by the dashed rectangle.

Scale bar: 1000 μm (upper row), 200 μm (lower row).

b: Number of MCH positive cells per sections at 45 (P45, n=6) or 110 (P110, n=4) days of age. ** *P* value<0.01, ANOVA followed by Tukey. Data are presented as mean and SEM.
